# Spread Through Air Spaces in Colorectal Lung Metastases Signals Local Recurrenece and Reflects Morphologic Aggressiveness of the Primary Tumor

**DOI:** 10.1111/pin.70107

**Published:** 2026-03-25

**Authors:** Taketo Nakai, Satoru Morita, Yutaka Kurebayashi, Masayoshi Monno, Ryo Seishima, Kohei Shigeta, Koji Okabayashi, Mari Mino‐Kenudson, Yuko Kitagawa, Keisuke Asakura

**Affiliations:** ^1^ Department of Surgery Keio University School of Medicine Tokyo Japan; ^2^ Department of Pathology Keio University School of Medicine Tokyo Japan; ^3^ Department of Surgery Inagi Municipal Hospital Tokyo Japan; ^4^ Department of Surgery Fujita Health University Toyoake Japan; ^5^ Department of Pathology Massachusetts General Hospital Boston Massachusetts USA

**Keywords:** pulmonary metastases, STAS, tumor budding grade

## Abstract

Tumor spread through air spaces (STAS) is a histological feature associated with poor prognosis in primary lung cancer, but its relevance in colorectal cancer (CRC) pulmonary metastases remains unclear. This study evaluated the prognostic impact of STAS in CRC pulmonary metastases and its association with histologic features of the primary tumor. A total of 124 patients who underwent pulmonary resection for CRC metastases were retrospectively analyzed. Quantitative STAS parameters, including density and maximum spread distance, were assessed histologically. Survival outcomes were analyzed using Kaplan–Meier and Cox proportional hazards models, and logistic regression identified predictors of STAS. STAS was present in 33.1% of patients and was associated with significantly shorter 5‐year recurrence‐free survival (18.7% vs. 53.0%, *p* = 0.002) and overall survival (*p* = 0.001). Quantitative STAS metrics correlated with intrathoracic recurrence. Patients with high tumor budding grade had a significantly higher STAS‐positive rate than those with none or low grade (60% vs. 31.2%, *p* = 0.036). Tumor budding independently predicted STAS (odds ratio: 3.19, 95% confidence interval: 1.05–9.69, *p* = 0.040). STAS independently predicted poor prognosis, particularly intrathoracic recurrence. Quantitative STAS assessment enhanced prognostic precision, and tumor budding grade may serve as a preoperative marker for predicting STAS.

## Graphical abstract

STAS in colorectal cancer (CRC) pulmonary metastases was significantly associated with worse overall survival and higher recurrence rates, particularly intrathoracic recurrence. Furthermore, this study showed that high tumor budding grade in the primary CRC was significantly correlated with the presence of STAS in the pulmonary metastases.

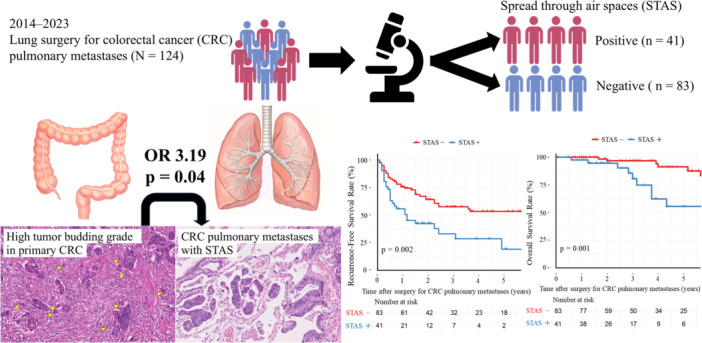

**Abbreviations**
BMI: Median body mass index
CEA: Carcinoembryonic antigen
CRC: Colorectal cancer
EMT: Epithelial–mesenchymal transition
OS: Overall survival
RFS: Recurrence‐free survival
STAS: Spread through air spaces

## 1 Introduction

Pulmonary metastases are frequent and clinically significant manifestations of advanced colorectal cancer (CRC) [1, 2, 3]. Recurrence after surgical resection remains frequent, highlighting the need for robust prognostic markers [4, 5]. One such marker gaining increasing attention is tumor spread through air spaces (STAS), a histological pattern initially identified in primary lung adenocarcinomas and subsequently associated with poor prognosis [6, 7]. STAS is now recognized in the World Health Organization classification of lung tumors [7].

Although STAS has been validated as an adverse prognostic factor in primary lung cancer [8, 9], its relevance in pulmonary metastases, particularly those of colorectal origin, remains largely unexplored. A few small cohort studies suggested that STAS in metastatic lesions may be linked to an increased risk of local recurrence [10, 11]. Moreover, the biological nature of STAS has been debated: it may be an artifact after surgical manipulation or sectioning [12, 13], but several molecular and immunohistochemical studies have supported its role as a true *in vivo* phenomenon [14]. For example, STAS‐positive non‐small cell carcinomas have demonstrated unique gene expression profiles, including upregulation of hypoxia‐responsive genes and chemokines, such as CXCL8 [15, 16]. Furthermore, STAS may be associated with tumor microenvironment alterations, including immune cell infiltration and extracellular matrix remodeling [17, 18]. Tumor‐associated macrophages and neutrophils have been implicated in promoting a permissive alveolar niche for pulmonary metastasis [19].

Despite these insights, most previous studies have assessed STAS as a binary variable (present vs. absent), and the prognostic impact of quantitative STAS metrics, including density and maximum spread distance, has not been systematically evaluated in CRC pulmonary metastases [20]. Moreover, the potential predictors of STAS in pulmonary metastases remain unclear. Primary tumor histologic features, including tumor budding and differentiation grade, may provide insights into the metastatic patterns and STAS risk.

This study aimed to evaluate the qualitative and quantitative impacts of STAS on recurrence‐free survival (RFS) and overall survival (OS) in the largest cohort of patients with CRC pulmonary metastases. Moreover, we sought to identify the clinicopathologic features predicting STAS and validate the prognostic utility of continuous STAS metrics using advanced statistical modeling techniques.

## 2 Materials and Methods

This retrospective cohort study was conducted on patients who underwent lung surgery for CRC pulmonary metastases between 2014 and 2023 at Keio University, School of Medicine. This study was approved by the institutional review board, and written informed consent was obtained from the participants.

### 2.1 Patient Selection

In total, 164 patients with CRC pulmonary metastases who underwent therapeutic lung resection between 2014 and 2023 were retrospectively reviewed. Patients were excluded if they had undergone lung surgery for biopsy, had margin‐positive (R1/R2) resection, had insufficient clinical data, or if STAS evaluation was unfeasible due to inadequate tissue or poor slide quality. Finally, 124 patients were included.

### 2.2 Surgical Strategy

The surgical approach for pulmonary metastases from CRC was determined based on tumor size, anatomical location, number of metastatic lesions, and patient‐related factors, with the primary goal of achieving complete resection while preserving pulmonary function. Wedge resection was preferentially selected for small peripheral lesions, generally measuring approximately ≤ 2–3 cm in maximum diameter, located within the outer one‐third of the lung parenchyma, provided that adequate surgical margins could be secured. For larger tumors, lesions located more centrally than the outer one‐third of the lung, or cases with multiple metastatic nodules within the same lobe requiring en bloc resection, segmentectomy was selected according to the tumor location. Lobectomy was reserved for cases in which complete resection with adequate margins was considered difficult to achieve by segmentectomy.

In patients with bilateral pulmonary metastases, the decision to perform a single‐stage or staged bilateral resection was individualized. Single‐stage bilateral surgery was considered for patients with preserved pulmonary function and low surgical risk, taking into account age, comorbidities, and overall functional status. In contrast, staged bilateral resection was selected for patients with reduced respiratory reserve or higher perioperative risk. In staged procedures, resection of the lung with a greater tumor burden or larger dominant lesion was generally prioritized. Furthermore, for patients initially planned for staged bilateral resection, treatment strategies were formulated to allow flexibility in subsequent management. In cases where postoperative pulmonary function deteriorated substantially after the first operation, alternative treatment modalities, including radiotherapy, were considered instead of proceeding with contralateral lung surgery. Nevertheless, all patients required bilateral resection achieved R0 resection in this cohort.

### 2.3 Histopathologic Evaluation

Formalin‐fixed paraffin‐embedded tissue sections from the resected pulmonary metastases and the corresponding primary CRC were reviewed independently by experienced pathologists, who were blinded to the clinical outcomes. Surgical margin distance was recorded by converting the stapled resection margin to an equivalent distance of 5 mm. STAS was defined as the presence of tumor cell clusters within air spaces in the lung parenchyma at a distance of at least 500 μm from the edge of the main tumor, according to established diagnostic criteria [10]. Interobserver concordance was 95.1%, with discrepancies resolved through consensus discussion. For patients with multiple metastatic nodules, STAS was considered positive if any nodule exhibited STAS. Among those nodules, the nodule with the greatest extent of STAS was used for determining the number and maximum linear distance from the tumor border, using a microruler. STAS density is the number of discrete STAS tumor cell clusters per square millimeter in the alveolar space, measured on hematoxylin‐and‐eosin‐stained sections. The primary tumor pathology was reviewed for tumor budding, lymphovascular or perineural invasion, and histological grade. Tumor budding was defined as the presence of isolated single tumor cells or small clusters composed of fewer than five tumor cells infiltrating the stroma at the invasive front. Tumor budding was assessed in a hotspot area using a ×20 objective lens, and cases were classified as none or low grade budding when 0–4 buds were identified and as high grade budding when five or more buds were present by experienced pathologists [21].

### 2.4 Clinical and Pathologic Variables

Demographic and clinicopathologic data, including age, sex, smoking status, carcinoembryonic antigen (CEA) levels before lung surgery, number and size of pulmonary nodules, RFS, and OS, were extracted from the electronic medical records.

### 2.5 Classification of Recurrence Sites

To analyze the recurrence patterns, the recurrence sites were classified as intrathoracic or extrathoracic and analyzed according to STAS status. Intrathoracic recurrence occurs within the thoracic cavity, including pulmonary parenchyma, pleura, and mediastinal lymph nodes. Extrathoracic recurrence occurs at any site outside the thoracic cavity, such as intraabdominal organs.

### 2.6 Statistical Analysis

Continuous variables were compared using Wilcoxon rank‐sum test, whereas categorical variables were compared using Chi‐square or Fisher exact test, as appropriate. Survival probabilities were estimated using Kaplan–Meier analysis and compared using log‐rank test. Multivariate Cox proportional hazards models were constructed to identify the independent prognostic factors. Additionally, the presence of STAS was analyzed as a continuous variable using STAS density and STAS maximum distance. A restricted cubic spline curve was employed to explore potential nonlinear relationships among STAS distance, density, and recurrence risk. All statistical analyses were performed using R version 4.2.0 (R Project for Statistical Computing, Vienna, Austria), with a two‐sided *p* value of < 0.05 indicating statistical significance.

## 3 Results

### 3.1 Patient Characteristics

The final analysis included 124 patients with a median age at surgery of 68.0 years (IQR: interquartile range, 59.0–74.0 years) and a median body mass index (BMI) of 23.0 kg/m^2^ (IQR, 20.0–26.0 kg/m^2^); 53.2% were female. The primary tumor was located in the colon and rectum in 62 patients each (50.0% each). The predominant histological subtype was well‐ to moderately differentiated adenocarcinoma (tub1/tub2; 97.6%). The median diameter of pulmonary metastases was 1.2 cm (IQR, 0.9–1.6 cm), with single and multiple lesions observed in 79 (63.7%) and 45 patients (36.3%), respectively. Synchronous and metachronous metastases were observed in 23 (18.5%) and 101 (81.5%) patients, respectively. Bilateral metastases were present in 21 patients (16.9%). Of these, 14 patients underwent single‐stage bilateral resection, whereas seven patients underwent staged bilateral resection. In all patients treated with staged bilateral resection, contralateral lung resection was performed within 4 months after the initial lung surgery, and complete (R0) resection was achieved. STAS was identified in 41 patients (33.1%) but was not histologically evident in 83 patients (66.9%). In patients with multiple metastatic lesions, including metachronous tumors, STAS positivity was the presence of STAS in at least one of the evaluated nodules. Details of the clinicopathological features of the cohort are summarized in Table 1.

**Table 1:** Clinicopathological characteristics of the study patients.

Variables	Category	Overall (*n* = 124)
Age, years (median [IQR])		68.0 [59.0, 74.0]
BMI (median [IQR])		23.0 [20.0, 26.0]
Sex (%)	Female	66 (53.2)
	Male	58 (46.8)
Smoking history (%)	No	55 (44.4)
	Yes	69 (55.6)
CEA before lung surgery, ng/mL (median [IQR])		2.3 [1.6, 3.9]
Location of primary CRC (%)	Colon	62 (50.0)
	Rectum	62 (50.0)
Histology of primary CRC (%)	tub1/tub2	121 (97.6)
	por	2 (1.6)
	muc	1 (0.8)
Size of primary CRC, cm (median [IQR])		4.0 [3.1, 5.5]
Tumor stage of primary CRC (%)	T1	5 (4.0)
	T2	9 (7.3)
	T3	82 (66.1)
	T4	28 (22.6)
Lymph node metastases of primary CRC (%)	No	56 (45.2)
	Yes	68 (54.9)
Vascular invasion of primary CRC (%)	No	27 (21.8)
	Yes	97 (78.2)
Lymphatic invasion of primary CRC (%)	No	52 (41.9)
	Yes	72 (58.1)
Perineural invasion of primary CRC (%)	No	45 (51.7)
	Yes	42 (48.3)
INF of primary CRC (%)	a	2 (2.2)
	b	87 (95.6)
	c	2 (2.2)
Tumor budding grade of primary CRC (%)	None/Low	32 (56.1)
	High	25 (43.9)
Chemotherapy before lung surgery	No	50 (40.3)
	Yes	74 (59.7)
Surgical procedure for pulmonary metastases (%)	Wedge resection	91 (73.4)
	Segmentectomy	13 (10.5)
	Lobectomy	20 (16.1)
Size of pulmonary metastases, cm (median [IQR])		1.2 [0.9, 1.6]
Number of pulmonary metastases (%)	Single	79 (63.7)
	Multiple	45 (36.3)
Laterality of pulmonary metastases (%)	Unilateral	103 (83.1)
	Bilateral	21 (16.9)
Metachronous pulmonary metastases (%)	Synchronous	23 (18.5)
	Metachronous	101 (81.5)
STAS (%)	No	83 (66.9)
	Yes	41 (33.1)

*Note:* *Missing values are excluded from the analysis.

Abbreviations: BMI, body mass index; CEA, carcinoembryonic antigen; CRC, colorectal cancer; INF, infiltrative growth; STAS, spread through air spaces.

### 3.2 Prognostic Impact of STAS on Recurrence and Survival

**Figure 1:**
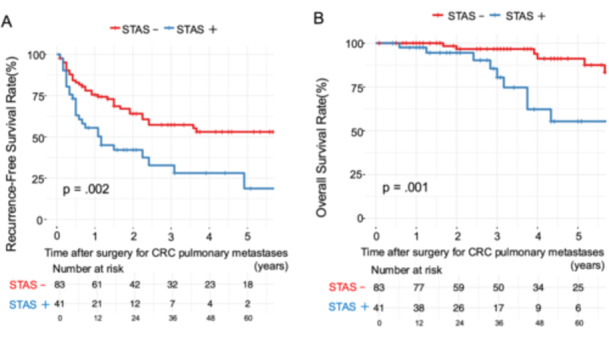
Comparison of Kaplan–Meier survival curves between STAS‐positive and STAS‐negative groups. After lung surgery, (A) RFS and (B) OS were significantly worse in the STAS‐positive group compared to the STAS‐negative group (*p* = 0.002 and *p* = 0.001, respectively; log‐rank test). Shaded areas represent 95% confidence intervals. Risk tables below each graph indicate the number of patients at risk over time. CRC, colorectal cancer; OS, overall survival; RFS, recurrence‐free survival; STAS, spread through air spaces.

The median interval between primary CRC resection and lung surgery was 24.2 months (IQR, 11.7–37.5 months). During a median follow‐up of 37.9 months (IQR, 21.8–61.7 months) after lung surgery, 21 (51.2%) and 26 (31.3%) patients in the STAS‐positive and ‐negative groups, respectively, developed intrathoracic recurrence. Kaplan–Meier analysis (Figure 1) demonstrated significantly shorter RFS in the STAS‐positive group than in the STAS‐negative group (5‐year RFS: 18.7% vs. 53.0%, log‐rank *p* = 0.002). Similarly, OS was significantly lower in the STAS‐positive group (5‐year OS: 55.3% vs. 91.2%, *p* = 0.001). Notably, multivariate Cox proportional hazards analysis revealed that STAS positivity (HR 1.85, 95% CI 1.08–3.17, *p* = 0.024) and size of pulmonary metastases (HR 1.47, 95% CI 1.07–2.01, *p* = 0.017) were independently associated with reduced RFS, conforming the prognostic relevance of STAS (Table 2).

**Table 2:** Univariate and multivariate Cox proportional hazards regression analyses for recurrence‐free survival.

	Univariate		Multivariate	
Variable	HR [95% CI]	*p* value	HR [95% CI]	*p* value
Age	0.99 [0.97–1.02]	0.615		
Sex: male	1.27 [0.77–2.10]	0.343		
Location of primary CRC: rectum	1.19 [0.72–1.96]	0.503		
Tumor stage of primary CRC: T4	1.82 [1.01–3.28]	0.047	1.46 [0.79–2.71]	0.230
Lymph node metastases of primary CRC: yes	1.36 [0.80–2.32]	0.251		
Chemotherapy before lung surgery: yes	1.07 [0.64–1.79]	0.788		
CEA before lung surgery	0.98 [0.89–1.07]	0.599		
Size of pulmonary metastases	1.53 [1.14–2.06]	0.005	1.47 [1.07–2.01]	0.017
Number of pulmonary metastases: multiple	2.02 [1.22–3.34]	0.006	1.62 [0.96–2.73]	0.069
STAS: yes	2.16 [1.30–3.58]	0.003	1.85 [1.08–3.17]	0.024

Abbreviations: CEA, carcinoembryonic antigen; CRC, colorectal cancer; STAS, spread through air spaces.

### 3.3 Clinicopathological Comparison Between STAS‐Positive and STAS‐Negative Groups

Table 3 summarizes the clinicopathological characteristics of STAS‐positive and STAS‐negative groups. There were no statistically significant differences in age (*p* = 0.328), BMI (*p* = 0.576), sex (*p* = 0.567), smoking history (*p* = 0.127), or primary tumor location (*p* = 0.409) between the two groups. Similarly, tumor stage (T1–3 vs. T4, *p* = 0.111), lymph node metastasis (*p* = 0.123), vascular (*p* = 0.648) and lymphatic (*p* = 0.563) invasion, histological subtype (*p* = 0.686), preoperative chemotherapy (*p* = 0.238), and surgical procedure for pulmonary metastases (*p* = 0.363) showed no significant association with STAS status.

**Table 3:** Comparison of the clinicopathological features between STAS‐positive and STAS‐negative groups.

Clinicopathological characteristics	Overall (*n* = 124)	STAS‐negative (*n* = 83)	STAS‐positive (*n* = 41)	*p* value
Age (median [IQR])	68.0 [59.0, 74.0]	70.0 [60.5, 74.0]	66.0 [58.0, 73.0]	0.328
BMI (median [IQR])	23.0 [20.0, 26.0]	22.0 [20.0, 26.0]	23.0 [21.0, 25.0]	0.576
Sex (%)				0.567
Female	66 (53.2)	46 (55.4)	20 (48.8)	
Male	58 (46.8)	37 (44.6)	21 (51.2)	
Smoking history (%)				0.127
No	55 (44.4)	41 (49.4)	14 (34.1)	
Yes	69 (55.6)	42 (50.6)	27 (65.9)	
Location of primary CRC (%)				0.409
Colon	62 (50.0)	40 (48.2)	22 (53.7)	
Rectum	62 (50.0)	43 (51.8)	19 (46.3)	
Tumor stage of primary CRC (%)				0.111
T1–3	96 (77.4)	68 (81.9)	28 (68.3)	
T4	28 (22.6)	15 (18.1)	13 (31.7)	
Lymph node metastases of primary CRC (%)				0.123
No	56 (45.2)	42 (50.6)	14 (34.1)	
Yes	68 (54.8)	41 (49.4)	27 (65.9)	
Vascular invasion of primary CRC (%)				0.648
No	27 (21.8)	17 (20.5)	10 (24.4)	
Yes	97 (78.2)	66 (79.5)	31 (75.6)	
Lymphatic invasion of primary CRC (%)				0.563
No	52 (41.9)	33 (39.8)	19 (46.3)	
Yes	72 (58.1)	50 (60.2)	22 (53.7)	
INF of primary CRC (%)^a^				0.271
a	2 (2.2)	2 (3.6)	0 (0.0)	
b	87 (95.6)	52 (92.9)	35 (100.0)	
c	2 (2.2)	2 (3.6)	0 (0.0)	
Tumor budding grade of primary CRC (%)^a^				0.036
None/Low	32 (56.1)	22 (68.8)	10 (40.0)	
High	25 (43.9)	10 (31.2)	15 (60.0)	
Histology of primary CRC (%)				0.686
tub1/tub2	121 (97.6)	81 (97.6)	40 (97.6)	
por/muc	3 (2.4)	2 (2.4)	1 (2.4)	
Chemotherapy before lung surgery				0.238
No	50 (40.3)	37 (44.6)	13 (31.7)	
Yes	74 (59.7)	46 (55.4)	28 (68.3)	
CEA before lung surgery (median [IQR])	2.3 [1.6, 3.9]	2.5 [1.6, 3.7]	2.1 [1.6, 5.0]	0.797
Size of pulmonary metastases (median [IQR])	1.2 [0.9, 1.6]	1.2 [0.9, 1.6]	1.2 [0.9, 1.5]	0.922
Surgical procedure for pulmonary metastases (%)				0.363
Wedge resection	91 (73.4)	64 (77.1)	27 (65.9)	
Segmentectomy	13 (10.5)	8 (9.6)	5 (12.2)	
Lobectomy	20 (16.1)	11 (13.3)	9 (22.0)	
Laterality of pulmonary metastases (%)				0.317
Unilateral	103 (83.1)	71 (85.5)	32 (78.0)	
Bilateral	21 (16.9)	12 (14.5)	9 (22.0)	
Number of pulmonary metastases (%)				0.238
Single	79 (63.7)	56 (67.5)	23 (56.1)	
Multiple	45 (36.3)	27 (32.5)	18 (43.9)	
Metachronous pulmonary metastases (%)				0.326
Synchronous	23 (18.5)	13 (15.7)	10 (24.4)	
Metachronous	101 (81.5)	70 (84.3)	31 (75.6)	

Abbreviations: BMI, body mass index; CEA, carcinoembryonic antigen; CRC, colorectal cancer; INF, infiltrative growth; STAS, spread through air spaces.

^a^ Missing values are excluded from the analysis.

### 3.4 Anatomical Pattern of Recurrence Among Patients Stratified by STAS Status

**Figure 2:**
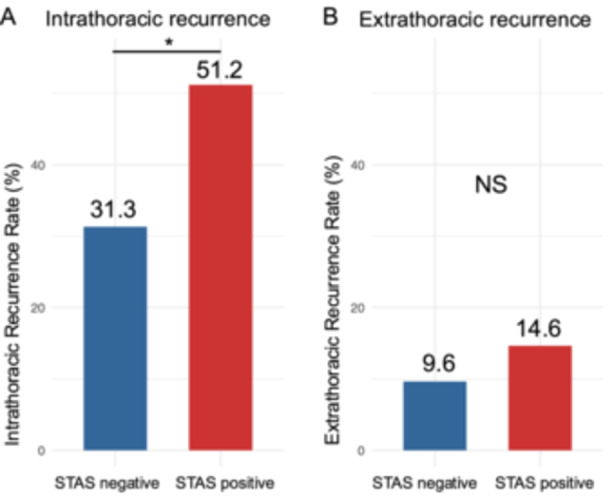
Bar plots of recurrence rates after lung surgery for CRC pulmonary metastases, stratified by STAS status. (A) Intrathoracic recurrence rate was significantly higher in the STAS‐positive group (*p* = 0.048), while. (B) extrathoracic recurrence rate did not differ significantly between groups (*p* = 0.550), based on Fisher's exact test. CRC, colorectal cancer; STAS, spread through air spaces.

The STAS‐positive group had significantly higher intrathoracic recurrence rate than the STAS‐negative group (51.2% vs. 31.3%, *p* = 0.048; Figure 2A). However, the extrapulmonary recurrence rate did not significantly differ between the two groups (*p* = 0.550; Figure 2B).

### 3.5 Resection Margin and Intrathoracic Recurrence

Among the 104 patients who underwent limited resection (wedge resection or segmentectomy), resection margin distance was compared between the STAS‐positive group (*n* = 32) and the STAS‐negative group (*n* = 72). The median resection margin distance was 11 mm (IQR, 9.3–16.5 mm) in the STAS‐positive group and 13 mm (IQR, 10.0–17.8 mm) in the STAS‐negative group, with no statistically significant difference between the two groups (*p* = 0.257). Using a cutoff value of 10 mm, which has been commonly considered an adequate resection margin for pulmonary metastases in previous surgical studies [2, 22], approximately three‐quarters of patients in both groups achieved a resection margin of ≥ 10 mm. The proportion of patients with a margin distance of < 10 mm did not differ significantly (*p* = 1.000) between the STAS‐positive (*n* = 8, 25.0%) and STAS‐negative groups (*n* = 19, 26.4%). We further assessed the association between resection margin distance and intrathoracic recurrence among the 104 patients who underwent limited resection. Using a 10‐mm cutoff commonly applied in pulmonary metastasectomy studies, intrathoracic recurrence occurred more frequently when margins were < 10 mm (40.5% vs 28.6%; *p* = 0.018). Within the < 10 mm subgroup, recurrence tended to be more frequent in STAS‐positive than STAS‐negative cases (87.5% vs 47.1%; *p* = 0.087).

### 3.6 Patterns of Intrathoracic Recurrence According to STAS Status

Among 47 intrathoracic recurrences (21 STAS‐positive and 26 STAS‐negative), staple‐line recurrence occurred exclusively in 3 STAS‐positive patients. The clinicopathological characteristics of the three cases with staple‐line recurrence are shown in Supporting Table 1. Same‐lobe recurrence occurred in three STAS‐positive (14.3%) and seven STAS‐negative patients (26.9%), whereas different‐lobe recurrence occurred in 14 (66.7%) and 17 (65.4%) patients, respectively. Mediastinal lymph node recurrence occurred in one patient (4.8%) in each group, and pleural recurrence occurred in 1 (3.8%) STAS‐negative patient only.

### 3.7 Quantitative Features of STAS and Correlation With Recurrence

Quantitative analysis of STAS characteristics revealed that STAS distance was significantly correlated with recurrence rate. Notably, the overall recurrence rates increased progressively with STAS extent, reaching 85.7% in the patients with STAS distance of > 1,000 µm (Supporting Figure 1A). Moreover, intrathoracic recurrence was observed in only 31.3% of patients without STAS but occurred in 50.0% and 57.1% of patients with STAS distances of ≤ 1,000 and > 1,000 µm, respectively (Supporting Figure 1B). The Cox proportional hazards model revealed clear dose‐dependent associations of recurrence risk with STAS distance and density (Supporting Figure 2).

### 3.8 Clinicopathological Features of Pulmonary Metastases and Corresponding Primary CRC Associated With STAS

**Figure 3:**
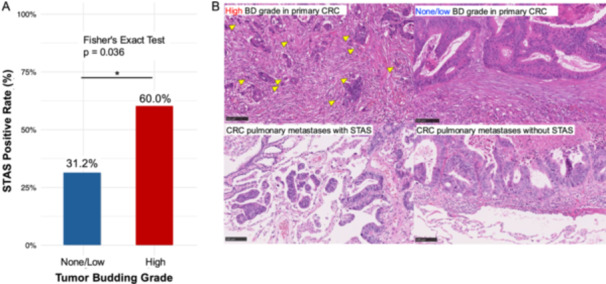
Association between tumor budding grade and STAS in CRC pulmonary metastases. (A) The bar graph shows a significantly higher STAS positivity rate in patients with high tumor budding grade compared to those with none or low tumor budding grade (60% vs. 31.2%, *p* = 0.036; Fisher's exact test). (B) Representative pathological images demonstrate features of high and none/low tumor budding grades in primary CRC, and the presence or absence of STAS in CRC pulmonary metastases (hematoxylin‐and‐eosin‐stained sections). CRC, colorectal cancer; STAS, spread through air spaces.

Age, sex, BMI, and the primary tumor location were not significantly different between the STAS‐positive and STAS‐negative groups. However, the STAS‐positive rate was significantly higher in patients with high tumor budding grade than in those with none or low tumor budding grade (60% vs. 31.2%, *p* = 0.036; Figure 3). Multivariate logistic regression analysis identified high tumor budding grade, a well‐established marker of aggressiveness and metastatic propensity　in CRC, as an independent predictor of STAS (OR: 3.19, 95% CI: 1.05–9.69, *p* = 0.040; Table 4).

**Table 4:** Univariate and multivariate logistic regression analyses for the predictors of STAS presence.

		Univariate		Multivariate	
Variables	Comparison	OR [95%CI]	*p* value	OR [95%CI]	*p* value
Age	Per 1 year increase	0.98 [0.95, 1.01]	0.273		
Sex: male	Female versus male	1.31 [0.62, 2.76]	0.486		
Primary CRC location: rectum	Colon versus rectum	0.76 [0.36, 1.61]	0.468		
Primary CRC tumor stage: T4	T1–T3 versus T4	2.58 [1.04, 6.38]	0.040	2.17 [0.58, 8.15]	0.251
Lymph node metastases of primary CRC: yes	No versus yes	1.89 [0.84, 4.23]	0.124		
Vascular invasion: yes	No versus yes	0.85 [0.33, 2.23]	0.746		
Lymphatic invasion: yes	No versus yes	0.72 [0.32, 1.60]	0.415		
Tumor budding grade: high (tumor budding 2–3)	None/low versus high	3.30 [1.10, 9.86]	0.033	3.19 [1.05, 9.69]	0.040
CEA before lung surgery	Per 1 ng/mL increase	0.95 [0.82, 1.09]	0.462		
Size of pulmonary metastases	Per 1 cm increase	1.26 [0.78, 2.03]	0.339		
Number of pulmonary metastases: multiple	Single versus multiple	1.62 [0.75, 3.50]	0.217		

Abbreviations: CEA, carcinoembryonic antigen; CRC, colorectal cancer.

## 4 Discussion

We investigated the clinical relevance of STAS in CRC pulmonary metastases, particularly focusing on its prognostic significance and association with the histopathological features of the primary tumor. Our results showed three principal findings. First, patients with STAS had significantly shorter RFS and OS than those without STAS, and STAS was confirmed as an independent adverse prognostic factor. Notably, the 5‐year RFS rate for patients with STAS was only 18.7%, underscoring its strong association with poor outcomes. Second, a quantitative relationship was observed between STAS burden and recurrence risk. Specifically, STAS density and maximum linear spread distance exhibited dose–response correlations with intrathoracic recurrence but not with extrathoracic recurrence. Third, primary colorectal tumor budding grade was a strong independent predictor of STAS positivity in metastatic lung lesions.

STAS is now recognized as an adverse prognostic factor in primary lung adenocarcinoma, particularly associated with locoregional recurrence after limited resection [23, 24]. Morphologically, STAS represents a form of aerogenous local spread in both lung adenocarcinoma and CRC pulmonary metastases, although whether this reflects shared biological mechanisms remains uncertain. Several hypotheses have been proposed to explain STAS formation in primary lung adenocarcinoma, including intrinsic invasive behavior, altered cell–cell adhesion, and interactions with the alveolar microenvironment; however, no definitive mechanism has been established [6, 14, 25]. In contrast, STAS in CRC pulmonary metastases represents the behavior of metastatic tumor cells that have already completed hematogenous dissemination and subsequently adapted to the alveolar architecture of the lung, potentially reflecting a secondary mode of local spread after implantation.

The evidence of its role in CRC pulmonary metastases remains scarce, with only a few small‐scale studies suggesting its clinical significance [10, 26] and none addressing the quantitative STAS characteristics. This study provided the largest cohort‐based analysis to date and uniquely incorporated a detailed quantitative assessment of STAS, including its density and extent. This quantitative approach was built upon a lung cancer study that reported worse outcomes with greater STAS extent [23, 27]. Notably, STAS distance and density were directly correlated with intrathoracic recurrence, supporting the hypothesis that STAS promotes localized dissemination through alveolar structures. This aligned with previous findings linking greater STAS burden to locoregional spread and recurrence risk in primary lung cancer [27].

Focusing on intrathoracic recurrence, particularly staple‐line recurrence, all three staple‐line recurrence cases identified in this study were observed exclusively in STAS‐positive tumors. Takeda‐Miyata et al. reported that the maximum spread distance of STAS in pulmonary metastases from CRC can reach up to approximately 11 mm, indicating the potential for tumor cell dissemination beyond conventional resection margins [10]. In our three cases, the resection margin distance was < 10 mm in all patients; however, each also demonstrated a relatively large STAS burden, characterized by extensive STAS spread distance and/or high STAS density (Supporting Table 1). These observations suggest that staple‐line recurrence may not be attributable solely to insufficient surgical margin, but that the extent and density of STAS may also contribute to microscopic tumor dissemination beyond the resection margin. Accordingly, STAS may represent not only a potential biological driver of recurrence but also a histologic marker of aggressive tumor behavior. From a clinical perspective, this observation suggests that STAS may have implications not only for further consideration of adequate resection margins, but also for the potential need to consider individualized postoperative management strategies in patients with CRC pulmonary metastases.

While tumor budding in primary CRC and STAS in pulmonary metastases share morphological features of tumor spread beyond the invasive front, we considered epithelial–mesenchymal transition (EMT) as one possible underlying mechanism, as previously implicated in both processes [25, 28, 29, 30]. However, our additional IHC (data not shown) in metastatic lung lesions did not demonstrate consistent EMT‐like changes—E‐cadherin was preserved and vimentin was absent—so the budding–STAS relationship should be interpreted as a statistical association rather than proof of a shared EMT program [31, 32, 33]. Quantitative STAS burden (distance and density) showed dose–response associations with intrathoracic recurrence, suggesting that, beyond serving as a marker of aggressive biology, STAS may contribute to local spread after implantation; nonetheless, causality cannot be inferred from this retrospective study. These observations support integrating STAS assessment into surgical planning and postoperative surveillance and motivate prospective, multi‐omic studies to define underlying mechanisms.

This study has several limitations. First, selection bias may have been introduced due to the retrospective, single‐center design. Second, histological assessment of STAS was inherently subjective and susceptible to interobserver variability. Third, more than half of the patients received chemotherapy, which may have acted as a confounding factor in the observed association between STAS and prognosis. Finally, the correlation of molecular or immunological markers with STAS, potentially yielding critical insights into its pathogenesis, was not evaluated. Future multicenter prospective studies incorporating genomic, transcriptomic, and spatial immune profiling are needed to validate our findings and uncover mechanistic pathways.

In conclusion, this study established STAS as an independent adverse prognostic factor in CRC pulmonary metastases and revealed primary CRC tumor budding grade as a significant predictor of STAS positivity. Our quantitative analysis of STAS burden provided additional prognostic information, particularly intrathoracic recurrence, supporting the integration of STAS evaluation into the clinical management of patients with CRC pulmonary metastases.

## Author Contributions

T. Nakai and S. Morita contributed equally to all aspects of this study. **T. Nakai:** Writing – original draft, methodology, investigation, formal analysis, data curation, conceptualization. **S. Morita:** Writing – original draft, methodology, investigation, formal analysis, data curation, conceptualization, supervision. **R. Seishima:** Writing – review and editing. **M. Monno:** Data curation, validation. **K. Shigeta:** Data curation, validation. **K. Okabayashi:** Data curation, validation. **Y. Kurebayashi:** Investigation, resources. **M. Mino‐Kenudson:** Writing – review and editing, conceptualization, supervision. **Y. Kitagawa:** Writing – review and editing, conceptualization, supervision. **K. Asakura:** Writing – review and editing, conceptualization, supervision.

## Conflicts of Interest

The authors declare no conflicts of interest.

## Supporting information


**Supplementary Figure 1:** STAS distance and recurrence rates.

**Supplementary Figure 2:** Linear correlation between STAS characteristics and recurrence rate.

**Supplementary Table 1:** Clinicopathological characteristics of the 3 cases with staple‐line recurrence.
