# Supplementary figures and images for: Spread Through Air Spaces in Colorectal Lung Metastases Signals Local Recurrenece and Reflects Morphologic Aggressiveness of the Primary Tumor
Source: Pathol Int. 2026 Mar 25;76(3):e70107. doi: 10.1111/pin.70107 (PMC13018295; doi:10.1111/pin.70107)

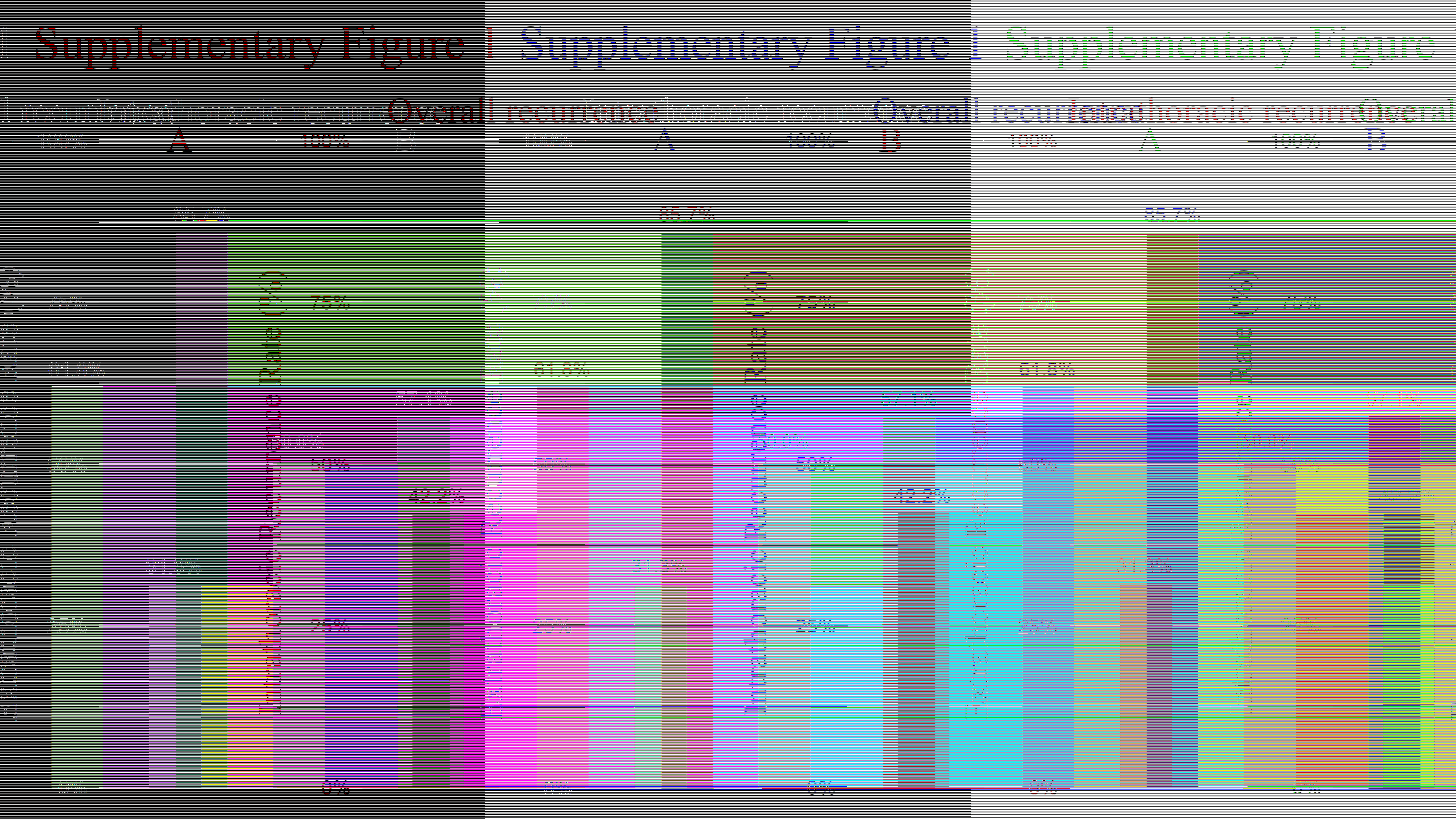

Supplement: Supplementary file 1 — Supplementary Figure 1: STAS distance and recurrence rates. [file PIN-76-0-s002.tif]

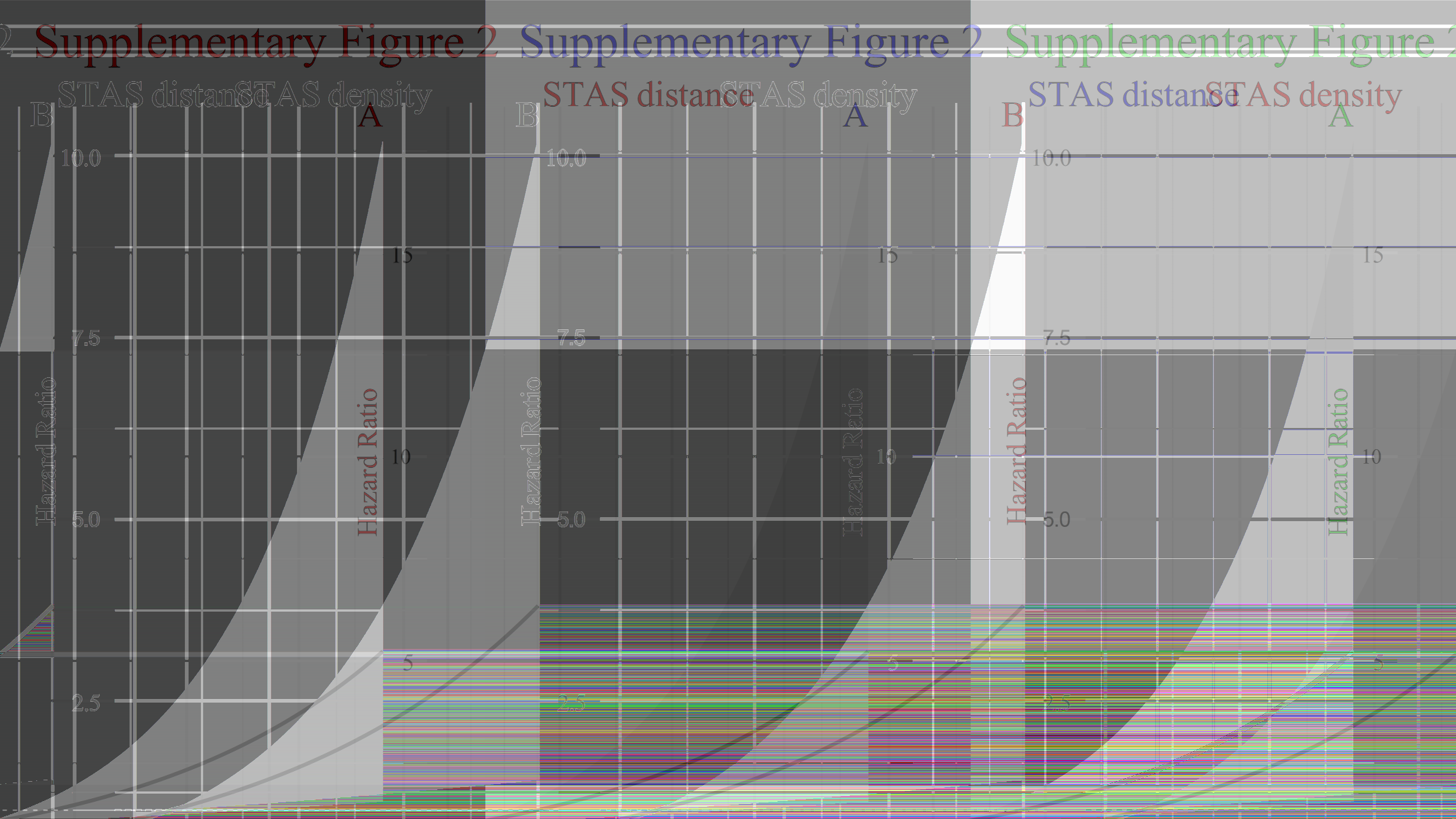

Supplement: Supplementary file 2 — Supplementary Figure 2: Linear correlation between STAS characteristics and recurrence rate. [file PIN-76-0-s001.tif]
