# Supplementary material for: Spread Through Air Spaces in Colorectal Lung Metastases Signals Local Recurrenece and Reflects Morphologic Aggressiveness of the Primary Tumor
Source: Pathol Int. 2026 Mar 25;76(3):e70107. doi: 10.1111/pin.70107 (PMC13018295; doi:10.1111/pin.70107)
Supplement: Supplementary file 3 — Supplementary Table 1: Clinicopathological characteristics of the 3 cases with staple‐line recurrence. [file PIN-76-0-s003.pdf]

Supplementary Table1. Clinicopathological characteristics of the 3 cases with staple-line recurrence

Case	STAS	STAS distance (μm)	STAS density (/mm²)	Tumor size (cm)	Surgical margin (mm)	Surgical procedure	RFS (months)	OS (months)	Dead or alive
1	Positve	1200	1	0.6	7	Wedge resection	3	29	Dead
2	Positve	550	4	0.8	8	Wedge resection	29	83	Alive
3	Positve	3500	10	1.5	6	Wedge resection	27	31	Alive

STAS: spread through air spaces, OS: overall survival, RFS: recurrence-free survival
